# The Phenotype List String Grammar for Enhanced Protein and Antigen Reporting in the Immunogenetic Context

**DOI:** 10.1111/tan.70693

**Published:** 2026-04-08

**Authors:** Steven J. Mack, Nicholas K. Brown, Loren Gragert, Jan A. Hofmann, William Lemieux, Benedict M. Matern, Kazutoyo Osoegawa, Jürgen Sauter, Martin Maiers, Eric Spierings

**Affiliations:** ^1^ Department of Pediatrics University of California San Francisco Oakland California USA; ^2^ Department of Pathology and Laboratory Medicine Perelman School of Medicine, University of Pennsylvania Philadelphia Pennsylvania USA; ^3^ Department of Pathology and Laboratory Medicine Tulane University New Orleans Louisiana USA; ^4^ DKMS Group Tübingen Germany; ^5^ Centre Hospitalier Universitaire (CHU) Sainte‐Justine Research Center Montréal Quebec Canada; ^6^ Department of Microbiology Infectiology and Immunology, University of Montréal Montréal Quebec Canada; ^7^ Center for Translational Immunology, University Medical Center Utrecht the Netherlands; ^8^ Research and Development, PIRCHE AG Berlin Germany; ^9^ Histocompatibility and Immunogenetics Laboratory Stanford Blood Center Palo Alto California USA; ^10^ CIBMTR (Center for International Blood and Marrow Transplant Research), NMDP Minneapolis Minnesota USA; ^11^ Matchis Foundation Leiden the Netherlands

**Keywords:** HL7 FHIR, HLA, phenotype list string, phenotype list string code

## Abstract

Standardised data representation is fundamental to the integrity, interoperability and reproducibility of immunogenetics and histocompatibility testing. While well‐established standards such as the genotype list (GL) String, histoimmunogenetics markup language (HML) and minimum information for reporting next‐generation sequence genotyping exist for encoding HLA genotyping results, no equivalent syntax currently exists for describing phenotype‐ or antigen‐level information that underlies antibody testing and immunological risk assessment. We introduce the *phenotype list* (PL) *String* grammar and the *phenotype list string code* (PLSC) syntax, structured, machine‐readable grammars designed to represent antigen‐ and protein‐level data across three contexts: (1) the HLA phenotype composition of assay reagents, (2) test results derived from those reagents and (3) clinical interpretations of HLA antibody specificities. PL String extends the hierarchical logic of the GL String grammar, while adapting a subset of GL String delimiters and rules to describe ambiguity, heterodimeric relationships and phenotypic composition. PL String incorporates explicit namespace definitions (e.g., World Health Organization HLA Nomenclature Committee for Factors of the HLA System, Organ Procurement and Transplantation Network, Eurotransplant and NMDP) to ensure traceability and compatibility with existing regulatory and operational frameworks. By harmonising how HLA phenotypic and antibody data are encoded, PL Strings enable automated data exchange between laboratories, registries and information systems, reducing transcription errors and improving computational interpretation. This new grammar establishes a foundation for interoperable representation of serologic and functional HLA data, facilitating reproducible research and enhancing clinical decision support in transplantation and immunogenetics. Technical specifications for PL Strings and PLSCs are available at plstring.org.

**Abbreviations**
ET: eurotransplant
FHIR: fast healthcare interoperability resources
GL: genotype list
GLSC: genotype list string code
HAML: HLA antibody markup language
HL: health level
HML: histoimmunogenetic markup language
JSON: javascript object notation
MAC: multiple allele code
OPTN: organ procurement and transplantation network
PL: phenotype list
PLSC: phenotype list string code
SAB: single antigen bead
WHO: World Health Organization nomenclature committee for factors of the HLA system

## 1 Introduction

Accurate and standardised representation of HLA data is a prerequisite for reliable data exchange and reproducible analysis, both in clinical practice and immunogenetics research [1]. The complexity and polymorphism of the HLA system [2] tremendously benefit from unambiguous, machine‐readable formats to ensure data integrity, support interoperability between systems and enable consistent interpretation across laboratories and institutions.

Several such standards have been established for genotyping in the immunogenetic context. Most notably, the genotype list (GL) String format [3, 4] provides a structured grammar to represent allelic and genotypic ambiguity in a consistent and standardised manner. GL Strings are important components of more detailed standards, such as GL String code [5], histoimmunogenetic markup language (HML) [6] and minimum information for reporting next generation sequence genotyping [7].

However, a comparable standard is lacking for antigen‐level HLA data, particularly in contexts where the focus shifts from genetic typing information to interpretation of serological or functional data (e.g., HLA antibody profiling [8] and cell‐based immunologic assays [9]). To address this need, we introduce the phenotype list string (PL String) format. PL Strings encode data that describe the HLA phenotype composition of reagents (e.g., cells or single antigen HLA proteins used in antibody detection assays), results of testing using such reagents, and clinical interpretations of such results (e.g., reporting of lists of acceptable and unacceptable antigens). The PL String syntax largely follows the GL String syntactic principles and elements, while some aspects of the syntax have been adapted to meet the semantic needs of phenotype‐ and antigen‐focused applications.

The PL String syntax has been developed in response to several challenges. First, there is currently no structured, widely adopted method for encoding lists of antigen‐level HLA data. Second, histocompatibility laboratories and vendors of antibody assays differ in how they document HLA protein‐based reagents used for antibody screening [10, 11]. Interpretation results on laboratory reports also vary in format, which complicates data exchange between laboratories and application by transplant programs, registries and allocation systems. This lack of HLA antibody data standardisation hampers automated analysis and increases the risk of misinterpretation when exchanging data across platforms and institutions.

Although nomenclatures for HLA alleles and antigens have existed for some time, their practical implementation in laboratory and vendor reporting has been highly heterogeneous. For example, an HLA‐DP antigen may appear as DP105 in one report, but this specificity may appear as DPB1*105:01 or DP‐0402 (etrl.eurotransplant.org/resources/hla‐tables/) in allocation systems. An interpretation of antibody assay data that identifies ‘84DEAV’ as an DPB1 unacceptable epitope may appear in a report of unacceptable antigens for the United States Organ Procurement and Transplantation Network (OPTN) systems, but other systems may not use this concept. Within a single report, inconsistent use of prefixes, leading zeros or delimiters further complicates interpretation. For example, both ‘C2’ and ‘C02’ are used, and lists of alleles and antigens may be separated by commas, spaces or both (e.g., ‘B7, B8’ versus ‘B7 B8’). Some laboratories may list a set of HLA specificities using a mixture of antigen‐level and non‐standard molecular HLA notations (e.g., a field labelled as ‘DR52’ in OPTN systems may contain ‘52’, ‘3*01’ and ‘3*03’ specificities). These inconsistencies in reporting HLA specificities illustrate the absence of both a shared grammar and a shared namespace across systems.

In addition to inconsistent terminology, there are no established standards for reporting ambiguity in assay definition or interpretation at the protein or antigen levels. Ambiguity is common in serologic or phenotype‐based assays. For example, when a serum analyte reacts with a set of phenotypic beads, each containing multiple HLA proteins, or when allele‐level uncertainty is not resolved in the definition of a reagent or cell (e.g., ‘DQB1*03:01/297’). Additional examples can be found in UCLA Health's Cell Exchange [9] reports (www.uclahealth.org/departments/pathology/research/research‐services/immunogenetics‐uic/services‐and‐pricing/reference‐programs/summary‐reports/cell‐exchange‐summary/cell‐exchange‐summary‐archive). Currently, laboratories use a variety of ad hoc notations (e.g., slashes, commas or parentheses) to indicate uncertainty, none of which are formally defined or interoperable.

A similar gap exists for the representation of heterodimeric HLA class II molecules such as HLA‐DQ, ‐DP or ‐DR, which consist of paired α‐ and β‐chains. Reports often describe these in free‐text form (e.g., ‘DQA1*05:01 with DQB1*02:01’) or using vendor‐specific symbols, making automated parsing or comparison across systems challenging. Isolated HLA‐DQ heterodimer reactivity against an HLA class II single antigen bead (SAB) may be represented on reports as ‘DQA1*05:01 ~ DQB1*02:01’, while other reports may represent these heterodimers as ‘DQA1*05:01/DQB1*02:01’, and many information systems may not recognise HLA heterodimer specificities at all.

Without a standardised format and syntax for HLA specificities involved in antibody assays, human interpretation of HLA data on reports remains necessary and interoperability between laboratories, software tools and registries in clinical, operational and research settings is severely limited.

It is important to note that the work described here, defining a format and syntax, focuses exclusively on the grammar and structure of data representation and not on revising or correcting the underlying HLA namespaces (e.g., World Health Organization HLA Nomenclature Committee for Factors of the HLA System [WHO] [12], EuroTransplant [ET] [13], OPTN [14], NMDP [15]) and nomenclatures (e.g., WHO molecular [hla.alleles.org/pages/nomenclature/naming_alleles/], WHO antigen [hla.alleles.org/pages/antigens/naming_hla_antigens/], NMDP multiple allele codes (MACs) [hml.nmdp.org/MacUI/#/], heterodimer molecules and OPTN DPB1 unacceptable epitopes) themselves. While we acknowledge the existence of best practices concerning the use of leading zeros, asterisks and other naming conventions, such issues fall outside the scope of the work described here. Accordingly, discussions on topics such as expression level characters have been deliberately omitted.

The PL String grammar introduces explicit rules and delimiters to encode both ambiguity and heterodimeric relationships in a structured, standardised manner. By addressing these challenges, PL Strings constitute a framework for harmonising HLA antibody reporting, enabling better traceability and improved clinical decision support. The PL String syntax directly addresses these issues by defining a clear, standardised syntax for representing HLA phenotypes, assay results and antibody specificities.

## 2 Design Principles

The PL String grammar builds on the foundational grammatical concepts established with the GL String grammar [4]. Both grammars are structured to be unambiguous and comprehensive to real‐world use‐cases while being human‐readable, machine‐parseable and hierarchical, ensuring clarity for end‐users and enabling automated processing and data integration. The guiding objective was to develop a distinct, yet semantically aligned, string grammar that addresses complementary use cases in the antigen‐centric domain of immunogenetics, be it for HLA or other genetic systems.

There are three distinct contexts for the use of the PL String grammar:

1. *Describing the phenotypic HLA antigen composition of a reagent or test system*. This can include the antigens expressed on a cell line, a bead set used in single‐antigen assays or a reference panel used in antibody screening. The PL Strings enable the identification of the HLA antigens presented to the immune system in the test context, independent of the underlying genotype.
2. *Describing a specific reagent or test applied to a particular sample, and yielding a certain test result*. To report such a result, PL Strings can refer to the reagent name alongside a ‘positive’, ‘negative’ or ‘inconclusive’ qualifier, indicating the sample's reactivity against that HLA antigen composition.
3. *Describing clinical interpretations made for HLA antibody specificities in patient samples*. PL Strings enable structured documentation of HLA antibody reactivity, with specificities considered present, acceptable or unacceptable. Interpretations may integrate information on serological reactivity from current and previous testing, clinical data about the patient's disease or treatment, prior sensitisation and previous transplants.

Although each PL String context addresses a distinct type of data (biological composition of reagents versus clinical interpretation of assays), they apply a shared grammar and notation to promote consistency, flatten learning curves, make use of synergies and simplify implementation in laboratory information systems and registries.

These distinct PL String contexts can be applied, in combination with GL String, for patient care. Consider a patient searching for a potential stem‐cell donor. HLA genotyping and SAB assays are performed to assess compatibility. The HLA genotyping assay result describes the patient's HLA genotype using a GL String. The specificity of the antigens on the beads that comprise the SAB panel is described using PL Strings. The patient's antibody specificities, determined by interpreting the results of SAB assays, are also described using PL Strings. Potential stem‐cell registry donors who carry any of the patient's HLA unacceptable antigens (also described using PL Strings) are excluded by default from the patient's search and match report. The genotypes of donors acceptable for that patient are described using GL Strings.

To address the requirements outlined above, it is essential to differentiate between the fundamental components (‘atoms’) of a PL String and the grammatical elements (‘delimiters’) that organise atoms into PL Strings. The atoms available for use within a PL String are determined by the selection of a specific namespace (Section 4.1), which defines the permissible elements. Examples of such namespaces include protein‐level HLA designations, as established by the IPD‐IMGT/HLA Database [16, 17, 18], and antigen mappings as defined by ET or OPTN. The definition and maintenance of each namespace's vocabulary fall under the exclusive authority of the respective governing body. Importantly, the specification of PL String grammar does not encompass the definition of these vocabularies; rather, it provides an explicit description of the delimiters and their role in constructing valid PL Strings.

## 3 Grammatical Atoms

### 3.1 Supported Resolutions, Namespaces and Nomenclatures

The PL String grammar can be applied in multiple contexts. Examples include a single protein (e.g., A*01:01 or DRB1*08:07), heterodimeric protein complexes (e.g., DPA1*01:03 ~ DPB1*06:01), protein ambiguities described using MAC codes (e.g., A*02:AB), broad antigens (e.g., A9) [19], antigen splits (e.g., A24) [19], associated antigens (e.g., A2403), broad match determinants (e.g., DQA05) and epitopes (e.g., Bw4). Nomenclatures that describe peptides presented by specific HLA molecules (e.g., T‐cell epitopes [20]) or structural motifs of HLA proteins that present specific peptides (e.g., T‐cell epitope groups [21]) are not supported.

### 3.2 String Delimiters and Precedence

As with GL Strings, PL String delimiters provide structural context to the grammatical atoms in a string. Table 1 describes the allowed delimiters for PL Strings and their applications, while Table 2 contrasts the delimiters and their operations for PL and GL Strings. Where the GL String grammar applies six delimiters, the PL String grammar applies only four – ‘+’, ‘/’, ‘~’ and ‘%’. Of these, the ‘%’ operator is unique to PL Strings and is applied only to antigens, as detailed below. Each operator is evaluated in the numerical precedence shown in Table 1.

**TABLE 1:** PL String delimiters.

Delimiter	Operation	Context	Precedence	Example	Additional information
+	AND	All	1	DPA01+DPB0402	Multiple molecules are present on a bead.
				A2+B7	Multiple reactivities are detected.
~	Heterodimer	All	2	DPA1*01:03~DPB1*06:01	Only two elements can be linked by a ‘~’.
%	Inclusive OR	Antigens	3	A2402%A2403	Logical ‘or’; non‐exclusive, meaning ‘either or both’.
/	Exclusive OR	Antigens	4	A0201/A0202	Exclusive ‘or’ (XOR), meaning ‘only one’. This delimiter's symbol and meaning are identical in a PL or GL String.
		Proteins		A*02:01/A*02:02	

*Note:* PL String delimiters are evaluated in the increasing numerical order shown in the precedence column. Delimiters can be applied in different application contexts, as detailed in the context and additional information columns.

**TABLE 2:** Application of GL and PL String delimiters.

Delimiter	Name	Operation	GL String	PL String
+	Plus sign	AND	YES	YES
/	Forward slash	Exclusive OR	YES	YES
~	Tilde	AND describing either chromosomal phase (GL String) or heterodimer membership (PL String)	YES	YES
%	Percent sign	Inclusive OR	NO	YES
^	Caret	AND (unphased individual loci)	YES	NO
|	Pipe	OR (genotypes)	YES	NO
?	Question mark	Locus ambiguity	YES	NO

*Note:* Ambiguity categories are represented via distinct delimiters in each of the ‘X’L String nomenclatures. In GL Strings and in PL Strings describing proteins, ambiguity regarding the presence of a specific variant is denoted with the ‘/’ delimiter, which indicates that only one of the variants in the slash‐delimited string is present. In PL Strings describing antigens, this ambiguity is denoted with the % delimiter, which indicates that any or all of the delimited variants may be present. The GL String and PL String columns identify which operators are used in a given ‘X’L String nomenclature.

### 3.3 Basic PL String Rules

Given that PL Strings can represent phenotypes, but not genotypes, they cannot identify a genetic locus, chromosomal phase, the cis/trans relationships between variants, or a specific chromosomal order of variants.

The PL String ‘+’ operator is applied to describe the components of assays and assay results, indicating that multiple molecules are present on a bead or that multiple reactivities have been detected in an assay.

The PL String ‘~’ operator identifies the elements of a heterodimeric protein, with no default or required order for subunits. For example, DPB1*02:02~DPA1*01:04 and DPA1*01:04~DPB1*02:02 are equally valid representations of a specific DP heterodimer. This ordinal‐freedom applies to all PL String delimiters. The PL String ‘~’ operator can only be flanked by the appropriate heterodimeric alpha and beta subunits or antigens, as defined by the chosen namespace and context (e.g., HLADQA1*05:01~HLA‐DQB1*02:01, SDQA05~DQ2).

The PL String ‘%’ and ‘/’ operators both indicate protein and antigen ambiguity, but differ in whether both specified proteins or antigens may be present. The ‘%’ operator indicates that either one or both antigens may be present, for example in the case of multiple HLA molecules on the same phenotypic bead. In contrast, the PL String ‘/’ operator can be used to represent ambiguity in the protein or antigen where the presence of both proteins or antigens is not expected (e.g., antigens on a bead).

Detailed examples of PL Strings, applied in multiple contexts, are provided in Table 3.

**TABLE 3:** PL String examples.

Example	Context	PL String	Description
HLA‐A phenotype list with ambiguous proteins	Reagent	HLA‐A*01:01/HLA‐A*01:02+HLA‐A*02:01	HLA‐A*02:01 is present. Either HLA‐A*01:01 or HLA‐A*01:02 are present, but both of them are not present.
HLA‐A antibody list with ambiguous proteins	Result	HLA‐A*01:01% HLA‐A*01:02+HLA‐A*02:01	HLA‐A*02:01 is present HLA‐A*01:01 and HLA‐A*01:02 might both be present or only one of them may be present.
HLA antibody list with OPTN antigen specificities		A2+A68+A69+B57+B58	Interpretation of antibody SAB data yields a list of 5 antigen‐level HLA specificities defined in the OPTN namespace, all of which are present.
Multi‐locus HLA antibody list		HLA‐A*01:02+HLA‐A*02:01+HLA‐B*07:02	All three, HLA‐A*01:02, HLA‐A*02:01, and HLA‐B*07:02 are present.
Multi‐locus HLA antibody list with heterodimer information		HLA‐DQA1*05:01~HLA‐DQB1*02:01% HLA‐DPA1*01:03~HLA‐DPB1*04:02	One or both of the heterodimers HLA‐DQA1*05:01~HLA‐DQB1*02:01 and HLA‐DPA1*01:03~HLA‐DPB1*04:02 are present.
Multi‐locus HLA antibody list with heterodimer information		HLA‐DPA1*01:04~HLA‐DPB1*02:01% HLA‐DPA1*01:04~HLA‐DPB1*04:02	One or two heterodimers are present, HLA‐DPA1*01:04 is always present; the second constituent is either HLA‐DPB1*02:01 or HLA‐DPB1*04:02.
		HLA‐DPA1*01:04~HLA‐DPB1*04:02% HLA‐DPA1*02:01~HLA‐DPB1*04:02% HLA‐DPA1*01:03~HLA‐DPB1*04:02	A heterodimer, in which HLA‐DPB1*04:02 is always present; the other constituent is either HLA‐DPA1*01:04, HLA‐DPA1*02:01 or HLA‐DPA1*01:03.
		HLA‐DPA1*01:04+HLA‐DPB1*04:01	Reactivity identified against two distinct protein specificities, rather than a heterodimer specificity.
		HLA‐DQA1*05:01~HLA‐DQB1*02:01+HLA‐DPA1*01:03~HLA‐DPB1*04:02	Two heterodimers are present.
		HLA‐DQA1*05:01~HLA‐DQB1*02:01/HLA‐DPA1*01:03~HLA‐DPB1*04:02	One of two heterodimers is present.
		HLA‐DPA1*01:04~HLA‐DPB1*04:02/HLA‐DPA1*01:03~HLA‐DPB1*04:02	One of two heterodimers is present, both include the HLA‐DPB1*04:02 subunit.

*Note:* An example of an HLA phenotyping reagent and eight examples of antibody interpretation results are shown. The specific interpretation of the operators in each PL String is provided in the description column. Operators are illustrated in green.

## 4 PL String Codes

A GL String is extended to a GL String code (GLSC) by appending identifiers for a gene family namespace and a code system version, delimited with hashtag (‘#’) symbols. PL String codes (PLSCs) are extensions of PL Strings that are similarly appended with namespace and code system identifiers, delimited with # symbols, as illustrated at plstring.org.

### 4.1 PL String Code Namespaces

To ensure unambiguous interpretation and reproducibility, each implementation of the PLSC syntax must clearly define the namespace from which its identifiers are drawn. A namespace represents the authority or organisation responsible for maintaining a specific controlled nomenclature or code system. Within the context of HLA‐related data, namespaces may correspond to regulatory, registry or institutional frameworks such as ET, NMDP, OPTN and WHO.

In addition to these four namespaces, any entity intending to define or extend a namespace must meet the following requirements:

1. *Governance*: The namespace must be maintained by a recognised organisation with a defined process for versioning, updates and public accessibility.
2. *Transparency*: The namespace definitions, including version identifiers and data sources, must be openly documented and traceable.
3. *Compatibility*: The namespace must adhere to the syntactic and semantic rules of the PL String syntax to ensure interoperability with other defined namespaces.
4. *Version control*: Each namespace implementation should reference a specific release version or date, following the principles of the IPD‐IMGT/HLA Database (www.ebi.ac.uk/ipd/imgt/hla/release/).

The ET, NMDP, OPTN and WHO namespaces enable representation of phenotype and antibody data in a manner consistent with existing regulatory and operational data exchange standards, as outlined and detailed below.

- *ET*: Codes and mappings used in Eurotransplant antigens and match determinants maintained by the ET Reference Laboratory (etrl.eurotransplant.org/resources/hla‐tables/) [22]. The ET namespace is recorded as ‘et’ in PLSCs.
- *NMDP*: Antigen and allele code definitions maintained for haematopoietic stem cell donor registries by NMDP https://hml.nmdp.org/MacUI/#/. The NMDP namespace is recorded as ‘nmdp’ in PLSCs.
- *OPTN*: Codes and antigen mappings used in U.S. organ allocation and virtual crossmatching (optn.transplant.hrsa.gov/policies‐bylaws/policies/). The OPTN namespace is recorded as ‘optn’ in PLSCs.
- *WHO*: The primary reference namespace for protein‐level HLA designations, maintained by WHO in the IPD‐IMGT/HLA Database (www.ebi.ac.uk/ipd/imgt/hla/). WHO provides authoritative, versioned definitions for HLA alleles and their corresponding proteins, which serve as the foundational vocabulary for PL String implementations at the protein resolution level. The HLA namespace is recorded as ‘who’ in PLSCs.

Of the four namespaces discussed above, the HLA namespace defines three levels of resolution relevant to PL String and PLSCs: protein level, antigen level and associated antigen level. Each level is detailed below. Application of PL Strings and PLSCs in these different contexts enables description of the reagents in an assay, the results of that assay and assay interpretation in a clinical setting.

#### 4.1.1 Protein Level

As described by Marsh et al. [2], 2‐field HLA allele names should be used for reporting unique proteins at the amino acid level. The allowed namespace is defined by the most recent IPD/IMGT‐HLA Database release. The HLA namespace allows explicit inclusion or exclusion of the ‘HLA‐’ prefix in 2‐field HLA assignments (e.g., ‘HLA‐B*07:02’ and ‘B*07:02’ are both allowed). Similarly, explicit inclusion or exclusion of expression variant suffices (A, C, L, N, S and Q) is also allowed in the HLA namespace (e.g., ‘A*24:09’ and ‘A*24:09N’ are both allowed).

#### 4.1.2 Antigen Level

Antigen categories predate molecular nomenclature and specify classes of HLA proteins that could readily be distinguished using serological testing methods. Only antigen designations published by WHO are allowed. Examples include B7, DR2, Bw4, Bw6, Cw9 and DPw2.

#### 4.1.3 Associated Antigen Level

Associated antigens are more recently refined antigen categories that were named based on a specific protein exemplar (e.g., the A2403 associated antigen is derived from the two‐field WHO allele A*24:03) and represent classes of proteins that were found to have similar reactivities against tested sera. The first set of associated antigen designations were published by WHO in 1991 [23], with more comprehensive sets of associated antigens published by Osoegawa et al. [24, 25, 26]. An expanded designation of associated antigens was incorporated into the HLA Nomenclature in 2026 [27]. Additional examples include B5102 and A‐0265; in this latter case, the inclusion of a dash (−) in the name of an associated antigen indicates that it has been proposed, but not published as part of the WHO nomenclature.

### 4.2 Implementation of PLSC in Communication Formats

Use of PL String information in communications requires specific conventions to ensure that the information remains interpretable, verifiable and interoperable across laboratories, databases and clinical information systems. Within structured exchange formats such as Health Level 7 (HL7) Fast Healthcare Interoperability Resource (FHIR) [28] and HLA Antibody Markup Language (HAML) [29] (github.com/immunomath/haml), the PLSC syntax must be used to represent alleles, proteins or antigens. This ensures that all identifiers are unambiguous, and that the reference database version can be identified without dependence on external metadata, enabling machine interpretation of PL String information over time. When multiple namespaces are included in a single record, each PL String code must specify the namespace it references. The mixing of identifiers from different namespaces within a single string is not permitted, as this would obscure provenance and semantic clarity.

If a specific IPD‐IMGT/HLA Database release version used for encoding cannot be specified, a date corresponding to the day the reagent was created, the results were produced or the interpretation was executed must be provided. This date must not refer to the date of sample collection or data export/extraction. In circumstances where there are multiple dates of reagent creation, result production or interpretation, separate PLSCs must be created for each date.

### 4.3 Exchanging PLSCs on HL7 FHIR Data Systems

**FIGURE 1:**
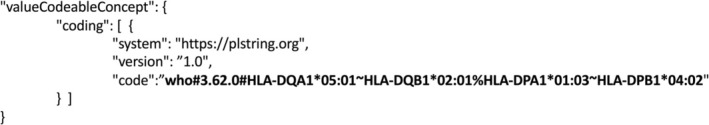
A phenotype list string code embedded in a compact JSON HL7 FHIR *valueCodableConcept*. In an HL7 FHIR message, the PLSC (shown in bold) is encapsulated within *valueCodableConcept* and *coding* tags. These define the code system (plstring.org) and the code system version (1.0).

The advent of PLSC enables the transmission of HLA phenotype and antigen data across HL7 FHIR systems [28] as elements of HL7 FHIR codable concepts. Laboratory results in HL7 FHIR Observation resources are embedded in *observation.valueCodableConcept* elements as the codable concept, in which the PLSC syntax is defined by the ‘plstring.org’ code system. HL7 FHIR systems using *valueCodableConcept* elements can wrap the PLSC in additional details of the code system and its associated version. A javascript object notation (JSON) version of a *valueCodableConcept* is illustrated in Figure 1.

### 4.4 Exchanging PLSCs Using HLA Antibody Markup Language

**FIGURE 2:**
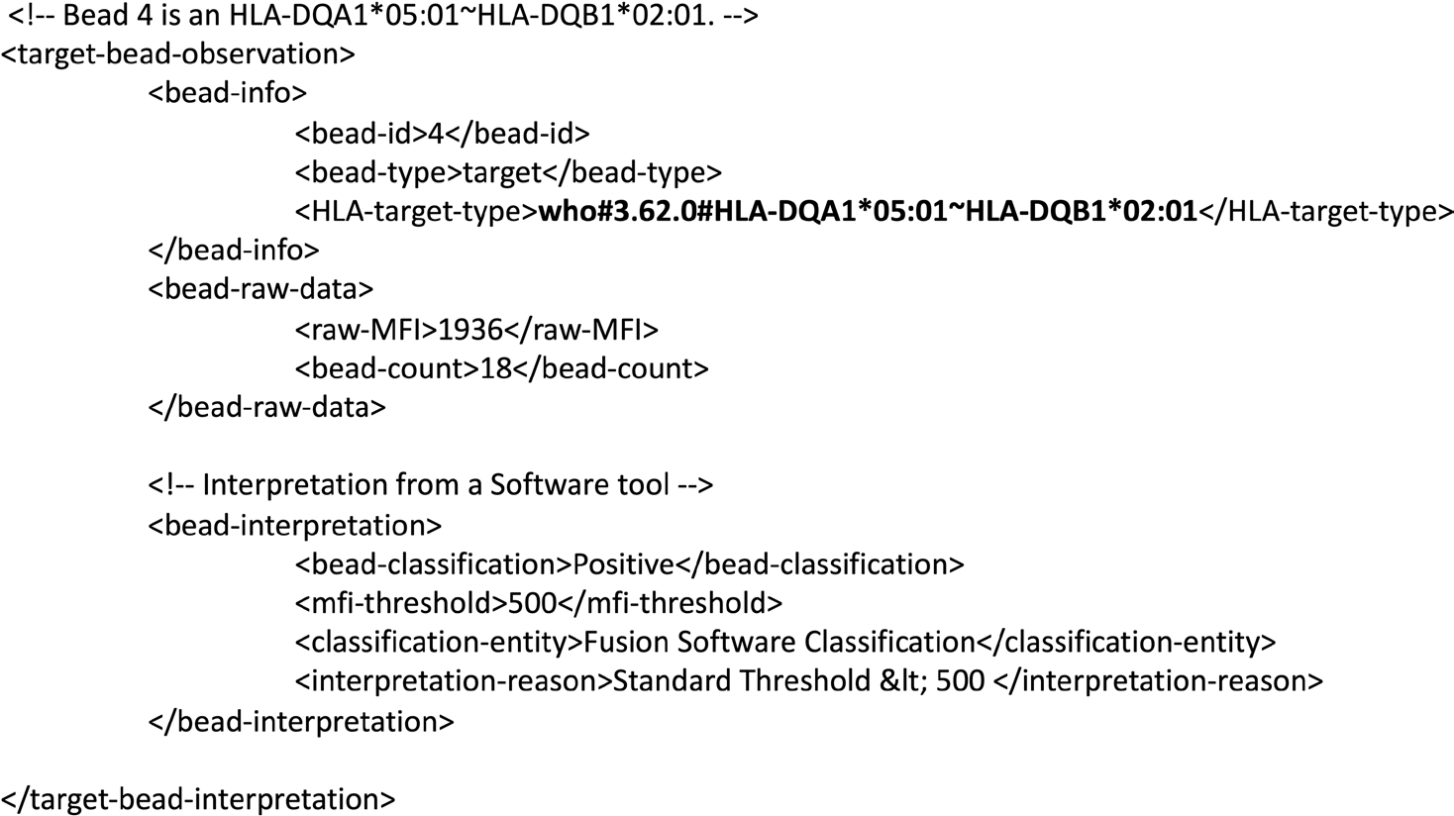
A phenotype list string code embedded in a HAML *target‐bead‐observation* element. In the *target‐bead‐observation* element of a HAML message, a PLSC (shown in bold) is encapsulated in an *HLA‐target‐type* element. The *bead‐raw‐data* and *bead‐interpretation* elements identify pertinent assay and interpretation data for that specific bead. Contextual identifiers are included between the ‘<!‐‐’ and ‘‐‐>’ comment delimiters.

PLSCs can also be exchanged as elements of HAML messages. HAML, an electronic data format standard for reporting HLA antibody assay data and interpretation results, is currently under development. HAML developmental version 0.5.3 allows the transmission of PLSCs for the use cases described in Section 2. In SAB assays, the specificity of a bead reagent can be described in a HAML message as a PLSC, as illustrated in Figure 2.

**FIGURE 3:**
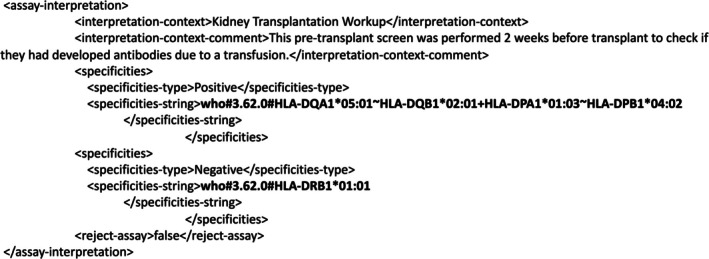
Phenotype list string codes embedded in a HAML *assay*‐*interpretation* element. Two PLSCs (shown in bold) are encapsulated in *specificities‐string* elements, nested within *assay‐interpretation* and *specificities* elements of a HAML message. Both positive (present) and negative (absent) antibody specificities for an assay interpretation are identified with PLSCs. Additional context for the assay interpretation is encapsulated within *interpretation‐context* and *interpretation‐context‐comment* elements.

Using human or computer‐based interpretation methods, individual beads are assessed for their reactivity against a specified antigen. The combination of multiple reagent‐specific reactivities is described in a PLSC representing positive or negative sample‐ or assay‐level reactivity, as illustrated in Figure 3.

**FIGURE 4:**
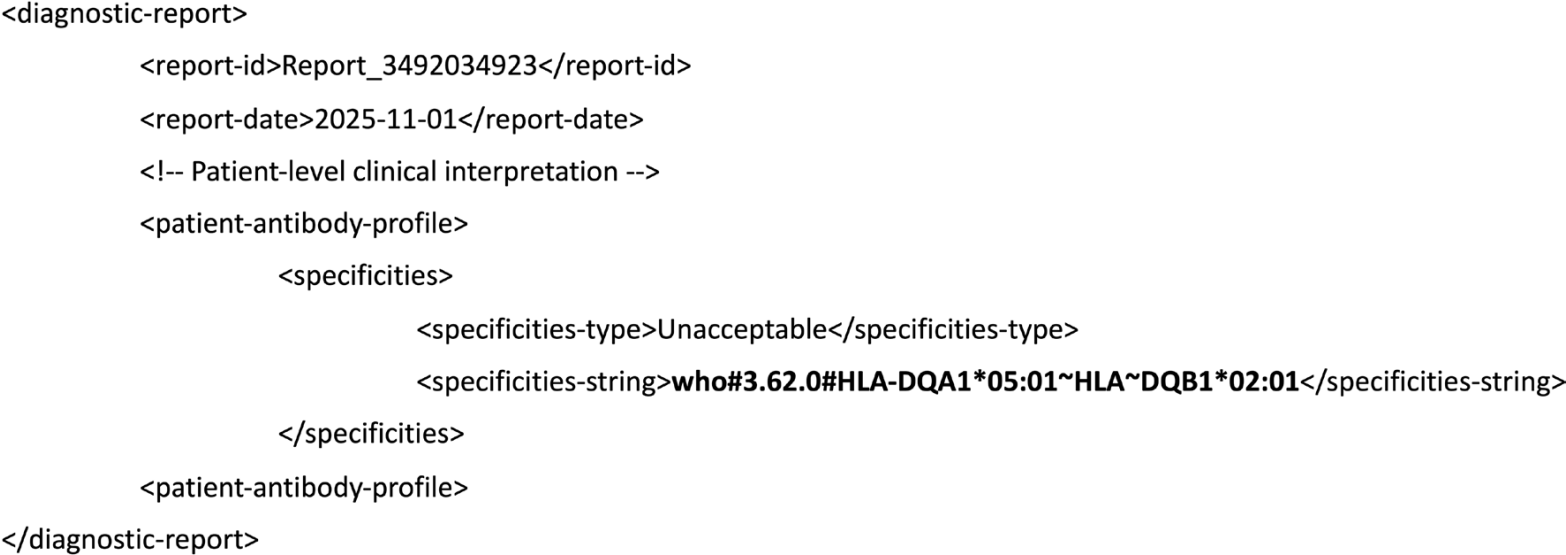
A phenotype list string code embedded in a HAML *diagnostic‐report* element. A PLSC (shown in bold) is encapsulated in the *specificities‐string* element within a *diagnostic‐report* element of a HAML message, as part of a patient antibody profile. The *specificities‐type* element identifies this PLSC as Unacceptable for the patient for whom this clinical interpretation was performed.

Finally, a PLSC can be applied as a clinical interpretation of an assay's results, for example, when antigen reactivity is interpreted to determine suitability for transplant, or to specify conditions for organ allocation. A PLSC specifying an ‘unacceptable antigen’, indicating that this antigen should not be present on a transplanted graft, is illustrated in Figure 4.

## 5 Discussion

The PL String grammar and its implementation as a PL String code establish a structural foundation that can accommodate future developments in immunogenetic data representation. Anticipated use cases for PL String and PLSC are presented in Table 4. Although the current specification focuses on protein‐ and antigen‐level information, the syntax is inherently flexible and can be extended to represent finer levels of molecular detail, once standardised nomenclatures become available (e.g., for eplets and epitopes).

**TABLE 4:** PL String and PLSC use cases.

Use cases	Likely syntaxes	Comments
Single antigen bead (SAB)	Protein‐level	Vendor worksheets indicate some protein‐level ambiguity in reagent definition (DQB1*03:01/297)
Flow crossmatches	Reagent (donor HLA typing or phenotype) paired with a positive/negative/equivocal qualitative assay result for T‐cell and B‐cell crossmatches	Deceased donors often have protein‐level typing ambiguity; HLA specificity typically cannot be determined from this type of assay.
(Un)acceptable antigens	Mixture	Current allocation system namespaces include multiple categories of syntactic atoms (antigens, two‐field alleles, epitopes).
Complement dependent cytotoxicity (CDC)	Reagent (donor HLA typing or phenotype) paired with a positive/negative/equivocal qualitative assay result	Deceased donors often have protein‐level typing ambiguity; HLA specificity typically cannot be determined from this type of assay.

*Note:* PL String and PLSC uses cases and their associated syntactic interpretations are described in four distinct contexts, with caveats for their interpretations provided in the comments column.

Beyond the classical HLA loci, the same grammatical principles can be applied to additional histocompatibility systems such as ABO, MICA, MICB and the non‐classical class I genes HLA‐E, HLA‐F, HLA‐G and HLA‐H. These loci increasingly feature in studies of transplant tolerance, immune modulation and disease association, yet currently lack harmonised data formats for phenotype or antigen reporting. Incorporating these grammatical principles within the PL String framework would provide a unified syntax for representing both classical and non‐classical histocompatibility molecules, thereby extending interoperability across broader immunogenetic domains.

The PL String design anticipates the evolution of HLA data standards toward greater molecular resolution. PL String's extensible structure ensures that future epitope‐based or non‐classical histocompatibility systems can be encoded, exchanged and interpreted using the same consistent grammar, preserving interoperability and backward compatibility with existing HLA genotype and phenotype data standards. For example, the HAML standard for representing and interpreting raw HLA antibody assay data is under active development and will be described in greater detail in future work.

The namespaces for some HLA categories remain non‐standardised and lacking in naming conventions. For example, eplet names may appear on reports issued by some histocompatibility laboratories, even though eplets are not yet used in allocation systems. The ‘62RR’ eplet refers to a different set of amino acid positions (62 and 65) than ‘62RN’ (62 and 63). Eplets that are composed of sets of noncontiguous amino acid positions are delimited by ‘+’ (which is also a PL String delimiter with the potential to confuse parsers), but these sets are not necessarily listed in position order (e.g., ‘62QE+56G’ and ‘43Q + 62GE’). Further, the descriptions of eplet names sometimes include parentheses that indicate certain additional positions may be involved. For example, the eplet ‘193PL’ includes a postulated position involved in antibody binding that is indicated in parentheses ‘193P194L(273S)’. To enable high fidelity integration of eplet nomenclature into PL Strings and PLSCs, organisations and teams that have governance over specific namespaces, such as the HLA Eplet Registry (www.epregistry.com.br) [30], should take on the task of developing clearly defined syntaxes, naming conventions and database versioning, and change management schemes for their respective nomenclature systems.

The PL String format has the flexibility to support future namespaces whose syntax and nomenclature have not yet been defined. For example, researchers in clinical immunology and histocompatibility continue to develop assays for T‐cell specificities using reagents composed of HLA proteins in complex with peptides. To communicate results for this category of assay data in a standardised way, a nomenclature to describe HLA tetramer reagent specificities and other assays involving HLA+peptide‐complex reagents is needed, and could apply many of the same grammatical atoms from the existing namespaces described here. A future nomenclature system for describing T‐cell specificities based on integrated interpretation across multiple immunologic assays and reagents has not been developed, but could be of use for pre‐transplant compatibility assessments and diagnosis of rejection after transplant in the future.

Aggregating HLA antibody data for multicenter or registry‐based studies has been logistically challenging due to inconsistency in reporting. Establishment of the PL String grammar is the first step toward addressing the current environment, in which tedious manual effort is required to organise datasets. We envision a future where information systems that support HLA data format standards incorporating PL Strings and PLSCs are interoperable. Consistent data specification is a critical prerequisite for development of computer‐assisted analysis tools that operate in a predictable and reproducible manner across assay types and histocompatibility information system platforms. PL String and PLSC will be made available for use through the collaboration of H&I researchers, clinicians, instrument manufacturers and software developers in future DaSH events, as was accomplished for the development of HML. Incorporation of PL Strings and PLSCs in research and laboratory information systems, as are GL Strings and GLSCs in HML, is the goal.

## 6 Conclusions

PL String is a novel, compact, standardised grammar that represents the antigen‐and protein‐level information in antibody assays and clinical interpretations. PL Strings and PLSCs complement GL Strings and GLSCs, enabling structured exchange of related phenotype and genotype data across laboratory and healthcare systems, for example as paired *valueCodableConcept* elements. As with GL String, the PL String grammar is agnostic with respect to the transmitted immunological vocabulary, ensuring its relevance for continually advancing nomenclatures and future immunotherapeutic methodologies.

## Author Contributions

All authors contributed to the conceptualisation of PL Strings and PLSCs and to the writing of the manuscript. S.J.M., M.M., L.G. and N.K.B. obtained funding support. S.J.M. drafted the final version of the manuscript, which was reviewed and approved by all authors. No artificial intelligence systems were applied in the writing of the paper or for the work described.

## Funding

This work was supported by National Institutes of Health, R01AI128775, R01AI173095.

## Ethics Statement

The authors have nothing to report.

## Conflicts of Interest

Authors Nicholas K. Brown, Jan A. Hofmann, William Lemieux, Benedict M. Matern and Jürgen Sauter affirm that they have no conflicts of interest to disclose with respect to the work described in this publication, the authorship of this publication or the publication of this article by the journal HLA. Loren Gragert is a member of the editorial boards of the journals HLA and Human Immunology and is a Section Editor for the journal Current Transplantation Reports. Steven J. Mack is a member of the editorial boards of the journals HLA and the International Journal of Immunogenetics and is an Associate Editor for the journal Human Immunology. Martin Maiers is a member of the editorial boards of the journals Cytotherapy, HLA, Human Immunology, Frontiers in Immunology and Frontiers in Genetics. Kazutoyo Osoegawa is a member of the editorial board of the journal HLA. Eric Spierings is an Associate Editor for the journal Frontiers in Immunology and a member of the editorial boards of the journals Cellular Immunology and HLA. These authors affirm that they have taken no actions to influence the peer review and/or publication of the work described and have no financial relationship with the journal HLA, its editor‐in‐chief or its publisher. They affirm that they have no financial conflicts of interests to disclose with respect to the work described, the authorship of this article or the publication of this article by the journal HLA.

## Data Availability

No new data were created for the work described in this publication. Details pertinent to PL String and PL String code are available at plstring.org.
